# Outcomes after Trifocal Femoral Fractures

**DOI:** 10.1155/2014/528061

**Published:** 2014-04-01

**Authors:** Michelle Griffin, Alastair G. Dick, Shamim Umarji

**Affiliations:** Orthopaedic Department, St. George's Hospital, London SW17 0QT, UK

## Abstract

Trifocal femur fractures are those of the femoral neck, diaphysis, and distal femur. These high-energy injuries predominantly occur in young people with the potential for long-term complications and disability. We present the cases of two men who were treated with proximal dynamic hip screws and distal periarticular locking plates to effectively manage trifocal femur fractures. Our cases have shown union at 2 years with good functional outcomes without the need for reintervention. We provide evidence for a successful surgical treatment option for these rare and complex injuries.

## 1. Introduction

Isolated fractures of the diaphysis of the femur are relatively common injuries with an estimated annual incidence of 10 per 100,000 person-years [1]. Multifocal fractures of the femur are less common with an additional proximal femoral fracture estimated to occur in up to 5% of diaphyseal fractures [2] and additional distal femoral fracture occurring in 3-4% [3]. Trifocal femoral fractures consisting of ipsilateral fractures of the proximal, diaphyseal, and distal femur are extremely rare. Such an injury pattern was first reported by Käch in 1993 [4] and to date the literature reports 18 cases of trifocal femur fractures [3–8]. These injuries result from high-energy mechanisms, usually a high-speed road traffic collision. Due to the rarity of such injuries and the heterogeneity of the fracture patterns there is minimal consensus on their optimal management. We present two cases of trifocal femur fractures managed with dynamic hip screws and distal periarticular locking plates, a technique which to the best of our knowledge has not previously been reported for the management of trifocal femur fractures.

## 2. Case Report 1

A previously fit and well 41-year-old male coach driver was involved in a high speed road traffic collision with a two-hour period of entrapment in his vehicle prior to extrication and transfer to our level 1 Trauma Centre. On arrival the patient was haemodynamically stable with no evidence of significant head injury. The patient was complaining of pain in the right thigh and had an obviously deformed right lower extremity.

**Figure 1:**
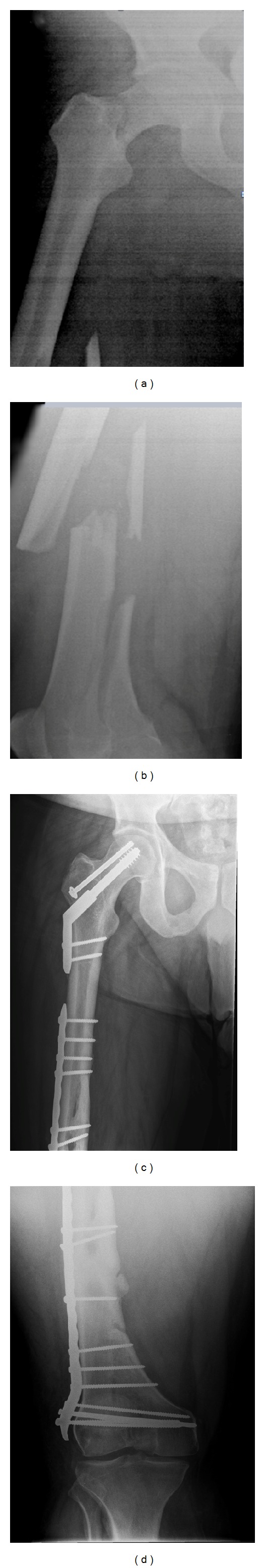
Case Report 1: (a)-(b) preoperative images, (c)-(d) postoperative images. The proximal fracture was reduced and stabilised using a two-hole plate dynamic hip screw with a derotation screw. An extended lateral approach was then used to reduce and stabilise the distal fractures with a 16-hole locking plate.

Radiographs revealed a grossly displaced diaphyseal femoral fracture with significant comminution and extension into the femoral condyles with a displaced ipsilateral basicervical femoral neck fracture (Figures 1(a) and 1(b)). The fractures were classified according to AO/ASIF classification as 31-B2, 32-B2, and 33-B2. The patient's other injuries included a right radius and ulna fracture and a subcapsular splenic haematoma, which was managed conservatively.

Surgery was performed on the same day, shortly after admission. Under general anaesthesia the patient was positioned on a standard radiolucent traction table. First the proximal fracture was reduced and stabilised using a two-hole plate dynamic hip screw (DHS) with a derotation screw. An extended lateral approach was then used to reduce and stabilise the distal fractures with a 16-hole locking plate (AxSOS Distal Lateral Femoral Plate, Stryker, Mahwah, NJ, USA) (Figures 1(c) and 1(d)). The upper limb injury was splinted but not definitively stabilised at this time.

The patient required an eight-day postoperative period in the intensive care unit. He developed acute renal failure secondary to rhabdomyolysis and required renal replacement therapy. He underwent an open reduction and internal fixation of the forearm fractures after five days. The patient then had an uneventful postoperative course and was discharged from hospital 23 days postoperatively.

At twelve-month follow-up there was radiographic evidence of complete union of the proximal fracture and evidence of some healing of the distal fractures. Clinically the patient had restricted movement of the hip and knee with pain on weight bearing. After 24 months the patient was pain-free with both distal and proximal fractures showing evidence of union with good alignment and position. He has required no further operative intervention; however, he has not yet returned to work.

## 3. Case Report 2

**Figure 2:**
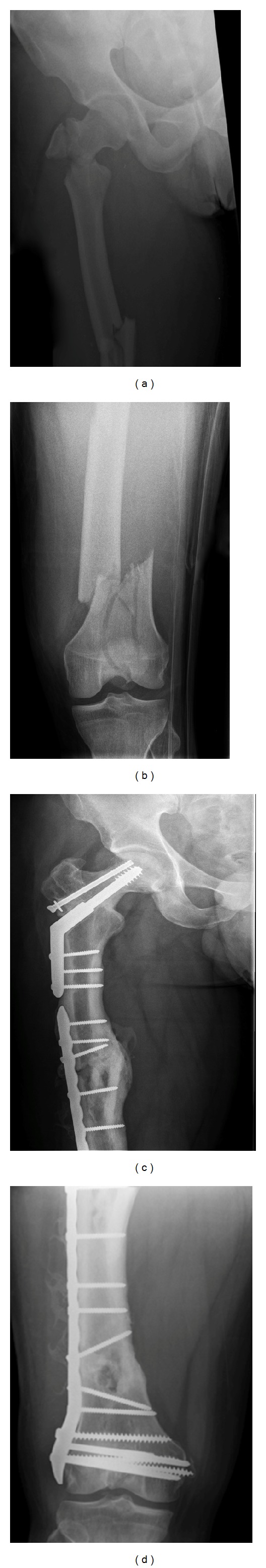
Case Report 2: (a)-(b) preoperative images, (b)–(d) postoperative images. The proximal fracture was reduced and stabilised with a three-hole plate and dynamic hip screw (DHS) with a derotation screw and a lateral approach was used to stabilise the distal femur fracture with a 14-hole locking plate.

A previously fit and well 47-year-old chef was a pedestrian involved in a road traffic collision. On arrival at our level 1 Trauma Centre the patient was complaining of pain in the right thigh and on examination had a shortened and external rotated lower limb. Radiographs revealed a right midshaft complex comminuted femoral fracture extending into the femoral condyles with an ipsilateral significantly displaced intertrochanteric femoral neck fracture (Figure 2(a)). The fractures were classified according to AO/ASIF classification as 31-A2, 32-B3, and 33-C1. The only other injury sustained was significant ligamentous damage to the contralateral knee.

Surgery was performed shortly after admission on the same day. The proximal fracture was reduced, closed, and stabilised with a three-hole plate DHS with a derotation screw. A lateral approach was used to reduce the distal fracture, which was then stabilised with a 14-hole locking plate (AxSOS Distal Lateral Femoral Plate, Stryker, Mahwah, NJ, USA) (Figures 2(b)–2(d)). Postoperative recovery was uneventful and the patient was discharged after 30 days.

Radiological union of the proximal fracture was evident at six months. At twelve months there was good callus formation at the distal diaphyseal-metaphyseal junction and the patient was able to partially weight bear with some pain. The diaphyseal fracture was the slowest to unite, with radiological union throughout the femur evident after 30 months. The patient's functional outcome has been excellent with a good range of pain-free movement at the hip and knee allowing him to return to work as a chef.

## 4. Discussion 

Trifocal fractures of the femur are difficult injuries to manage as the operative techniques and implants employed to manage one of the three fractures could compromise optimal management of the other fractures [3]. Basic principles suggest that a distal intra-articular fracture should be managed with anatomic reduction and rigid fixation producing absolute stability [9]. An intracapsular proximal fracture in a young patient also requires anatomic reduction for an optimal outcome [10]. Extracapsular proximal femur fractures can be fixed with relative stability, as can diaphyseal fractures [11, 12]. There is a wide range of operative techniques that could provide appropriate stability and fixation for the individual fractures; however, the difficulty lies in combining techniques to provide optimal fixation for all three fractures [3]. Previous reports have agreed that it is appropriate to use two implants only, with the diaphyseal fracture being stabilised with either the proximal or distal fracture [3, 6].

Given the rarity of trifocal fractures and the heterogeneity in fracture configurations there is little consensus in the literature as to what implants should be used and in what order stabilisation should take place [8]. The literature strongly suggests an individualised approach to the treatment of these injuries, as the heterogeneity of fracture configurations means treatment must be tailored to the individual personality of the fractures [3, 6–8]. Priority should be given to the injuries associated with worse outcomes if left untreated or mal-reduced [3].

A range of previous techniques have been described to manage the proximal component of trifocal fractures including the usage of cannulated screws, DHS, and intramedullary hip nails [3, 8]. Previously described techniques for the management of the distal fractures have included retrograde intramedullary nailing for extra-articular AO/ASIF type A fractures [3], cannulated screws for type B fractures [6, 7], and 95° blade plates for type C fractures [4].

We describe our experience of the successful management of two cases of trifocal femur fractures using a combination of a DHS to stabilise the proximal fracture and a periarticular locking plate to stabilise the distal and diaphyseal fractures together. Both cases have been followed up to 2.5 years and have shown full union, without significant complications. To the best of our knowledge this combination of implants has not previously been described in the literature.

The order of fixation for managing trifocal injuries has been a matter of debate [8]. Our cases suggest that good outcomes can be achieved with initial treatment of the proximal fracture, followed by fixation of the distal fracture as suggested by Barei et al. [3]. The poor outcome associated with delayed treatment of a distal femoral fracture is likely less than that of a proximal fracture with the potentially devastating complication of avascular necrosis of the femoral head [3]. However, as with the choice of implants, the order of fixation should be dictated by surgeon's preference, equipment availability, and fracture configuration [3, 8].

## 5. Conclusion

Trifocal femur fractures are extremely rare high-energy injuries and therefore all cases should be reported to allow management to be scrutinized and improved. We report a successful surgical outcome with the combination of a DHS for the proximal fracture and a periarticular locking plate for the distal fractures.
